# Prior thermal and high-pressure processing alters the impact of high intensity ultrasound on reconstituted skim milk

**DOI:** 10.1016/j.ultsonch.2024.107000

**Published:** 2024-07-22

**Authors:** Oluyemi Oriomah, Estefanía Noriega Fernández, Izumi Sone

**Affiliations:** a Department of Chemistry, Bioscience and Environmental Engineering, University of Stavanger, Richard Johnsensgate 4, 4021 Stavanger, Norway; b Department of Processing Technology, Nofima AS. Richard Johnsensgate 4, 4021 Stavanger, Norway; c European Food Safety Authority, Via Carlo Magno 1A, 43126 Parma, Italy

## Abstract

Reconstituted skim milk was subjected to heat treatment at 85 °C for 20 min or high pressure processing (HPP) at 400 or 600 MPa for 15 min with or without subsequent high intensity ultrasound (US) at 68 kHz, 500 W for 15 min at 30 °C. Untreated and treated samples were analyzed for particle size distribution, zeta potential, surface hydrophobicity, and concentration of total and surface sulfhydryl groups in addition to Native- and SDS-PAGE of serum phase upon ultracentrifugation and pH adjustment. Preceding heat- and HPP altered the impact of the subsequent US treatment, demonstrating process- and intensity-dependent exposure and burial of surface reactive sites on milk proteins respectively. US following HPP promoted sedimentation of HPP-dispersed serum casein fractions, while US following heat was directed mainly at the whey proteins originally bound to the micelles. The primary US effect on the untreated and treated milk proteins was at the molecular level.

## 1 Introduction

Potential of high intensity ultrasound (US) to enhance dairy protein structural and functional properties has gained research attention [1]. Previous studies have attributed US enhancing effect on milk protein gels to heat-induced whey protein denaturation [2], [3], size reduction of milk fat globules, increased fat-casein/whey protein interaction [4], breakup of casein-whey protein aggregates [5] and enhanced surface hydrophobicity [6], leading to higher gel firmness, increased viscosity and water holding capacities of resulting milk gel, as well as to the acceleration of gel formation [4], [7]. In addition, US effect will depend on sample composition such as presence of milk fat globules, casein to whey protein ratio [8], solvent [9] as well as US operational parameters, including power, frequency, sonication time and temperature [4], [6], [10].

Several authors have reported potential of thermosonication and manothermosonication (simultaneous application of heat and ultrasound under moderate pressure) to improve rheological and textural properties of dairy products [7], [11]. Nguyen and Anema [2] on the other hand observed only marginal effects of simultaneous heat and US treatment on skim milk, concluding that most of the effect was related to the heat generated during simultaneous heat and US treatment. Later Nguyen and Anema [3] found synergetic effects on milk gel properties in whole milk upon sequential heat (80 °C, for 30 min) and US (22.5 kHz, 50 W, 20 °C for 30 min) treatment and proposed that such additive effects were specific to whole milk with fat globules present. It is possible that their observation points to potential process-dependent effect: US effect on milk and acid gelation was influenced, in this case enhanced, by heat-induced changes prior to US treatment, not only by the presence of fat globules. While previous studies with US have mainly dealt with untreated sample, thermal as well as non-thermal milk processing such as high-pressure processing (HPP) at various pressure intensities are known to induce process-dependent changes to the milk proteins, as comprehensively reviewed by several authors [12], [13]. To this end, rreconstituted skim milk was thermally processed at 85 °C for 20 min or HPP treated at 400 or 600 MPa for 15 min, with or without subsequent US treatment at 68 kHz, 500 W for 15 min at 30 °C. In addition, untreated milk with and without sonication were considered, with the aim to assess and elucidate the potential process-dependent US effect on milk proteins as affected by prior thermal and non-thermal processing technologies.

## 2 Materials and methods

### 2.1 Preparation of reconstituted skim milk

Medium heat skim milk powder was kindly provided by TINE AS (Oslo, Norway). The milk powder contained 36.9 g protein and ∼0.8 g fat/100 g according to the manufacturer. Reconstituted skim milk was prepared by mixing milk powder with autoclaved distilled water to a final concentration of 130 g/L for 90 min on a magnetic stirrer (Hei-PLATE, Heidolph Instruments GmbH & Co. KG, Schwabach, Germany) at room temperature. The solid content of the reconstituted skim milk was 11.6 ± 0.051 % (N = 3) after drying at 105 °C for 20 h.

### 2.2 Milk processing

For heat treatment, the milk in a glass bottle was heat-treated in a beaker containing pre-heated water at ∼90 °C while constantly stirred to ensure uniform heat distribution during treatment. The water temperature in the beaker decreased upon introduction of the milk and the heat treatment for 20 min was started when the heating water temperature reached 85 °C. The heat-treated sample was immediately cooled in an ice bath to ∼30 °C. For HPP, the milk was transferred in a sous-vide polyethylene pouch (16 × 20 cm) (NorEngros AS, Stavanger, Norway) and vacuum-packed (Supermax C, Webomatic, Germany). HPP was performed at 400 or 600 MPa for 15 min by using a high hydrostatic pressure machine QFL 2L-700 (Avure Technologies Inc., Columbus, USA) pre-programmed at 410 or 610 MPa to ensure the actual pressure levels above 400 and 600 MPa throughout the holding time respectively.

Selected heat- and HPP-treated milk samples were immediately subjected to US treatment at 68 kHz, 500 W for 15 min in a glass bottle using BT 130H bench top ultrasonic water bath (35.56 cm W × 50.8 cm L × 35.56 cm H) (UPCORP, Illinois, USA). The initial water temperature of the US bath was maintained at 28 to 30 °C to ensure sample temperature at ∼30 °C upon competition of US treatment. The nominal specific power applied was 0.021 W/g.

### 2.3 Milk characterisation

#### 2.3.1 Particle size change and zeta potential

The untreated and treated milk sample was dispersed in filtered (0.22 µm) simulated milk ultrafiltrate (SMUF) at a volume ratio of 1:50, vortexed and filtered through a 0.8 µm Sartorius Minisart® Syringe filter (VWR international, Puerto Rico, North America) according to the method described by Jørgensen et al. [14]. 60 µL of the sample mixture was pipetted into ZEN2112 QS 3.00 mm cuvette (Malvern Panalytical, Malvern, UK). The measurement of particle size distribution was performed with Zetasizer Nano ZSP (Malvern Panalytical, Malvern, UK) at 25 °C with a He/Ne ion laser (λ = 633 nm) at 173° scattering angle and refractive index of 1.45. For the zeta potential measurement, 1 mL of the sample solution was injected into a Malvern disposable folded capillary Zeta Cell DTS1070 (Malvern Panalytical, Malvern, UK) prior to the analysis.

#### 2.3.2 Surface hydrophobicity

Surface hydrophobicity was determined by using fluorescent probe 1-anilinonaphthalene-8-sulfonic acid (ANS) as described by Alizadeh-Pasdar and Li-Chan [15] with modifications. The sample was diluted with 0.1 M phosphate buffer, pH 7.0 to a series of protein concentrations. 8 Mm ANS solution was prepared in 0.1 M phosphate buffer, pH 7.0 Sample and control solutions were transferred to a Hellma 104F-QS 10 mm size cuvette (Sigma-Aldrich, Wertheim, Germany) and the emission spectrum was read at 410–550 nm in Horiba Fluorolog-3 Modular Spectrofluorometer (Horiba Instruments, New Jersey, USA), with the excitation at 390 nm, with slit 2 nm/2 nm. The intensity at 470 nm obtained was used in calculating the relative fluorescence Intensity (RFI) as follow:

(4) $$\mathrm{RFI}=\Delta Int_{470}/ANS_{470}*Abitrary\;number$$

where ΔInt_470_ represents the difference between the intensity of the sample with and without ANS at 470 nm, ANS_470_ is the average intensity obtained for ANS in methanol and the arbitrary number of 30 was used for standardization. The resulting RFI was plotted as a function of concentration, and the slope of the graph was taken as the hydrophobicity index (Ho).

#### 2.3.3 Total and surface sulfhydryl (SH) content

The content of total and surface SH groups was determined according to Patrick and Swaisgood [16] and Ou et al. [17] with modifications. Sample and 2 mM DTNB (5,5-dithio-bis-(2-nitrobenzoic acid) were mixed with buffer 1 (86 mM Tris, 0.092 M Glycine and 4 mM EDTA, pH 8.0) and buffer 2 (8 M urea in buffer 1) for surface and total SH content respectively. Sample mixtures were incubated at room temperature for 30 min, (only for surface SH content) followed by ultracentrifugation at 45,000 rpm at 20 °C for 30 min using a Beckman Coulter Optima XPN-100 Ultracentrifuge with 70Ti rotor (Beckman Coulter, Inc, Indiana, USA) and filtration through a 0.45 µm Sartorius Minisart® Syringe filter (VWR international, Puerto Rico, North America). The supernatant was transferred to a 2.5 mL disposable macro cuvette (Sigma-Aldrich, Wertheim, Germany), and the absorbance was read at 412 nm using UV mini-1240 UV–VIS Spectrophotometer (Shimadzu, Kyoto, Japan). The content of total and surface SH groups was estimated based on [18]:

(3) $$\mu M\;SH/g=(73.53*\Delta Abs_{412}*D)/C$$

where ΔAbs_412_ denotes the difference in absorbance at 412 nm between the sample with and without DTNB, C is the protein content of the milk sample (47.97 mg/mL) calculated based on the information provided by the manufacturer and D is the dilution factor (2.2).

#### 2.3.4 Native- and sodium dodecyl sulfate (SDS) −polyacrylamide gel electrophoresis (PAGE)

∼15 g of the sample was transferred to a 38 mL thick wall polycarbonate centrifuge tube (Beckman Coulter, Inc. Brea, California) and ultracentrifuged at 100,000*g* at 20 °C for 1 h using an Optima XPN-100 Ultracentrifuge (Beckman Coulter, Inc. Indiana, USA) with Ti-70 rotor (Beckman Coulter, Co. Clare, Ireland) to obtain the supernatant with serum (non-sedimentable) protein fractions [19]. Another set of samples were subjected to pH adjustment to ∼pH 4.6 using about ∼150 µL 6 M HCl as described by Anema and McKenna [20]. The pH adjusted sample was centrifuged in a Thermo Fisher Scientific Heraeus Multifuge X3 FR centrifuge (VWR international, Oslo, Norway) at 3000 rpm for 30 min at 20 °C before the supernatant was retrieved. The protein concentration of the supernatant upon ultracentrifugation and pH adjustment was adjusted to 3.0 mg/mL with distilled water following semi-quantitative determination using NanoPhotometer (Implen GmbH, München, Germany) with distilled water as a blank.

Native-PAGE was run with the supernatant of ultracentrifuged and pH-adjusted samples after the respective sample solution was mixed with Native Sample Buffer (Bio-Rad, California, US). SDS-PAGE was performed with the ultracentrifuged sample by mixing the sample solution with 2x Laemmli sample buffer with ß-mercaptoethanol (Bio-Rad, California, US). The mixture was cooked using a Grant heating block QBT 4 at 100 °C (Grant instruments Ltd, England, UK) for 5 min and cooled at room temperature. For both Native- and SDS-PAGE, 30 µg protein was loaded into each well of 12-well Any kD Mini-PROTEAN TGX stain-free Precast Protein Gels with 10 µL of Unstained Precision Plus Protein Standard (Bio-Rad). The gel was run in Mini-Protean Tetra Vertical Electrophorese Cell at 300 V for 20 min with 1:10 running buffer (Bio-Rad, California, US). The gels were activated and imaged using ChemiDoc XRS+ (Bio-Rad) and analysed using Image Lab software (version 6.0.1, Bio-Rad).

#### 2.3.5 Statistical analysis

Significant effects of US were evaluated by running one-way ANOVA (with Tukey’s B as post hoc test) and *t*-test to compare sample to compare between treatments (i.e. untreated, heat-treated, HPP 400 MPa and HPP 600 MPa) and to determine effects of US treatment within each treatment in SPSS software (IBM, New York, US) version 26.0. Results with p < 0.05 were reported as significant.

## 3 Results and discussion

### 3.1 Particle size distribution and zeta potential

**Fig. 1:**
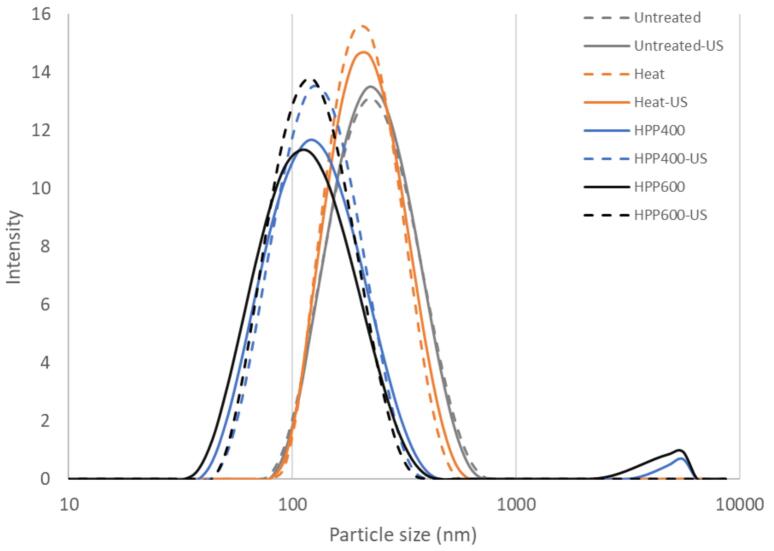
Representative graphs of particle size distribution of untreated and treated samples with (solid line) and without US (dotted line) diluted with SMUF at ratio 1:50.

The particle size distribution of the untreated and treated samples with and without subsequent US treatment is illustrated in Fig. 1. The untreated and heat-treated sample exhibited a monomodal distribution of particle sizes, with a slightly lower Z-average size of heat-treated sample (by ∼16 nm) when compared to that of the untreated (Table 1). This is in agreement with earlier studies [21], [22] where fewer denaturation of whey proteins at mild temperature limited increase in particle size as demonstrated at 80 °C for 30 min by Anema et al. [21].

**Table 1:** Average Z-average size and polydispersity index (PDI) with standard deviation for the untreated and treated samples. Values with different letters indicate significant differences (P < 0.05) between the treatment groups.

Treatment	Z-average size (nm)	PDI
Untreated	215.83 ± 1.87^a^	0.16 ± 0.01^a^
Untreated-US	212.87 ± 2.38^a^	0.13 ± 0.03^a^
Heat	199.80 ± 1.25^b^	0.14 ± 0.01^a^
Heat-US	200.27 ± 0.60^b^	0.11 ± 0.03^a^
HPP 400	117.77 ± 0.55^c^	0.22 ± 0.01^b^
HPP 400-US	119.67 ± 0.95^c^	0.14 ± 0.01^a^
HPP 600	109.37 ± 0.71^d^	0.21 ± 0.01^b^
HPP 600-US	113.20 ± 0.44^d^	0.14 ± 0.01^a^

Upon HPP at 400 and 600 MPa for 15 min, the major intensity peak of the respective samples was shifted to the left, resulting in a decreasing Z-average size with the increasing pressure intensity. The HPP-treated samples showed a bimodal distribution characterized by an intensity peak around ∼4500 to 4600 nm and the corresponding higher PDI value. Similar particle size reduction has been previously reported due to HPP-induced solubilization of micellar calcium phosphate, which in some cases may be accompanied by aggregation and bimodal size distribution [23], [24].

**Fig. 2:**
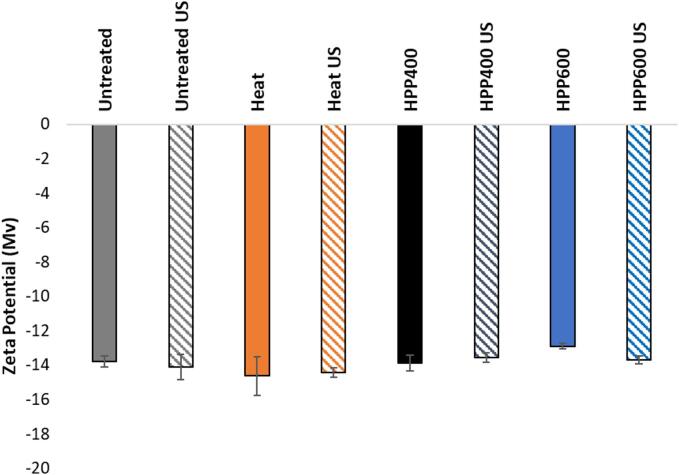
Average zeta potential (Mv) with standard deviation of untreated and treated samples with and without US.

With the subsequent US treatment, the bimodal size distribution of the HPP samples changed to monomodal, with a slight increase in the Z-average size and a lower PDI value. Similar size reduction by US has been attributed to cavitation shear forces at temperatures below denaturation temperature of whey proteins (<40 °C) [3]. However, no such significant effect of US was observed on the particle size distribution and Z-size average of the untreated and heat-treated sample, implying that US physical effect was largely limited to the HPP-induced aggregates of particle size ∼4500 to 4600 nm. US shearing forces are generally more effective in breaking up large multimeric protein complexes than individual monomeric proteins [11]. On the other hand, previous studies have demonstrated narrowed distribution and reduction of particle sizes by US (∼20–40 kHz) in untreated and (simultaneously or sequentially) heat-treated samples of dairy protein systems [2], [3], [7]. US physical effect is dependent on frequency in such that stronger shockwaves and microjets events are generated at lower frequency through fewer cavitations leading to more effective reduction in particle size [25], as opposed to a higher frequency as in this study (68 kHz) where main prevalent effects are chemical [26]. Relatively small changes in sample zeta potential (Fig. 2) indicated that the chemical or physical characteristics of the outer layer of the casein micelle was not affected during heat-, HPP- or subsequent US treatment in agreement with earlier studies [22], [27], [28].

### 3.2 Surface hydrophobicity

**Fig. 3:**
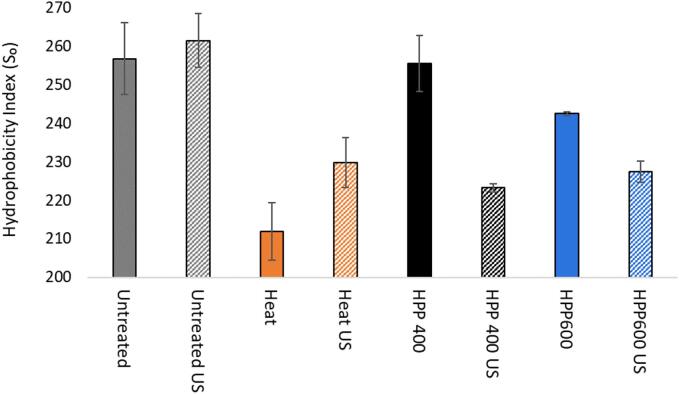
Average hydrophobicity index (S₀) with standard deviation of untreated and treated samples with and without US.

Heat treatment at 85 °C, 20 min significantly decreased sample surface hydrophobicity (Ho) (Fig. 3). The lower Ho in the heat-treated sample indicated that the mild heat treatment (85 °C, > 20 min) induced conformational changes and burial of hydrophobic sites in the heat treated sample while there was little evidence of subsequent increase in the particle size (Table 1). Only a negligible effect of HPP at 400 MPa on sample Ho was observed. This was unexpected considering the formation of large aggregates upon HPP 400 MPa, though minor in relative intensity (Fig. 1). The increased surface area of smaller particles (Fig. 1) generated by HPP-induced dissociation and dispersion [29] may account for the lack of HPP effect on Ho despite the concomitant formation of large aggregates. It could also be related to b-LG refolding after pressure release at HPP 400 MPa [30] within a few hours between the treatment and the analysis. However, the extent of refolding and subsequent effect on Ho is uncertain as it has been shown to vary by concentration and rather enhanced at lower protein concentration [13]. So is the potential interference of HPP on electrostatic interactions between the anionic probe ANS and the proteins [15], [31], as the sample zeta potential was shown to be unaffected by the treatment (Fig. 2). The results were similar to the minor and non-significant effect on Ho observed in HPP-treated plant proteins at high concentration (>4%, as in this study) [32].

The lower Z-average and size distribution upon HPP 600 MPa (Table 1; Fig. 1) showed the formation of significantly smaller particles hence increased surface area as argued for HPP 400 MPa. However, the lower Ho upon HPP 600 MPa in comparison indicated that the surface hydrophobic sites of these dispersed particles were less accessible by ANS. This could be due to more extensive denaturation and conformational change at the higher pressure, at which the refolding of β-Lg dimers at pressure release is hampered [13] and more stable hydrophobic and disulfide-linked interaction are formed [29].

Upon US, the Ho of untreated sample was little affected by US as observed by Chandrapala et al. [33]. However, the Ho of the heat-treated sample showed an non-significant, increasing trend (p = 0.063) by US, similar to those reported for various untreated and pre-treated protein systems [34], [35], [36], [37]. On contrary, the Ho of the sample treated at HPP 400 and 600 MPa decreased significantly upon the subsequent US treatment. Considering the lack of US effect on the Ho of the untreated sample, these results indicated that US effect was mainly directed at the thermally- and HPP-modified hydrophobic surface sites. However, the respective effects were opposite, demonstrating exposure and burial of surface hydrophobic sites following heat- and HPP treatments respectively.

### 3.3 Total and surface SH content

**Fig. 4:**
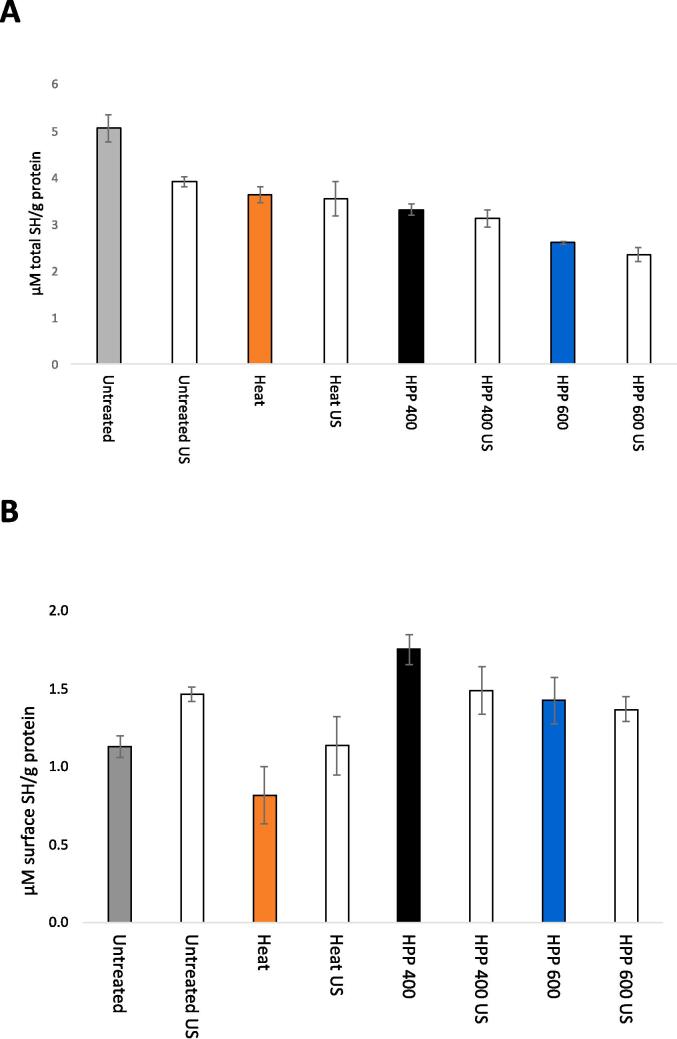
Average concentration of total (A) and surface (B) SH group (µM SH/g protein content) with standard deviation for untreated and treated samples with and without subsequent US.

The total SH content significantly decreased through heat treatment and HPP at increasing pressure intensities (Fig. 4) indicating process-induced disulfide bond formation. Heat treatment at 85 °C, 20 min showed a significant decrease in total and surface SH content indicating the formation of thermally induced disulfide bonds and conformational change that buried the respective reactive sites [38] with a minor effect on the particle sizes (Fig. 1, Table 1). Similarly, there was a significant decrease in the total SH content upon HPP 400 MPa indicating HPP-induced formation of sulfide bonds. On the other hand, the surface SH content significantly increased by HPP 400 MPa indicating heat- and HPP treatment induced different conformational changes to the surface reactive sites. The increased surface SH content upon HPP 400 MPa may be related to the unfolding and / or dispersion of reactive β-LG dimer as well as casein fractions, κ-CN and α-CN carrying two cysteine residuals [29]. HPP induced dispersion can expose αs2-CN which is originally not the surface component of the CN micelle [12]. The significantly lower total SH content upon HPP 600 MPa indicated more extensive denaturation and formation of more stable S-S bonds at the higher pressure where denatured β-LG, κ-CN, and α-CN as well as α-LA are incorporated [29], also corresponding with the lower surface SH content of the sample.

Upon US, there was a significant decrease in the total SH content of the untreated sample indicating that US promoted sulfide mediated interaction without leading to particle size change (Table 1; Fig. 1). Moreover, the concomitant significant increase in the surface SH group implied unfolding and increased exposure of buried SH groups in the untreated sample by US. These results corresponded with those reported by Shen et al. [36], [37] for untreated whey protein solutions subjected to US at 20 kHz. When following heat treatment, US induced no significant change in the total SH content while there was a non-significant, increasing trend in the surface SH content. Similarly, Shen et al. [36], [37] observed no significant change in total SH content by US treatment (>20 min) of pre-heated whey proteins, while the surface SH content increased along with the surface hydrophobicity.

US following HPP 400 MPa, 15 min showed a non-significant, decreasing trend in the surface SH content indicating possible conformation change and subsequent burial of surface reactive sites. There was little change in the total SH content by US following HPP 400 MPa, limiting the prevalence of new sulfide bond formation or disrupting the existing ones [36], [37]. Following HPP 600 MPa, US showed little effect on the total and surface SH content of the sample, on contrary to the significant decrease observed in the surface hydrophobicity by the US treatment (Fig. 3).

While US yielded some formation of new S-S bonds and interactions along with unfolding in the untreated sample (Fig. 3), the US effect in the heat- and HPP (400 MPa)-treated sample was mainly limited to the surface SH groups of native, native-like and/or thermally and HPP- altered structure without forming new disulfide bonds or disrupting exiting ones. The respective effects of US on surface SH content differed following heat- and HPP (400 MPa)- treatment, exposure (unfolding) and burial (renaturation) of surface SH groups respectively, corresponding to the process-dependent effects on surface hydrophobicity (Fig. 3). Additionally, the results indicated that HPP 600 MPa caused more extensive denaturation and conferred a higher stability of HPP-induced S-S bonds in the dispersed- and/or denatured proteins that were more difficult to disrupt by US, while the surface hydrophobic sites of the sample treated at HPP 600 MPa were more susceptible to US.

### 3.4 Native- and SDS-PAGE

**Fig. 5:**
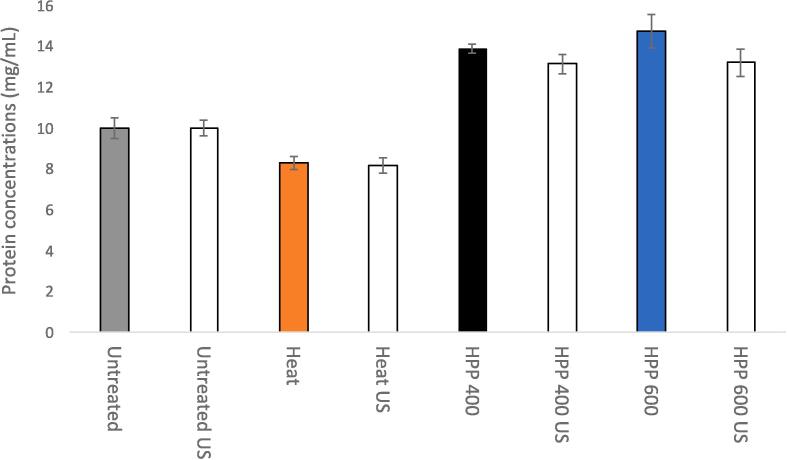
Average protein concentration in the serum phase of untreated and treated sample upon ultracentrifugation.

**Fig. 6:**
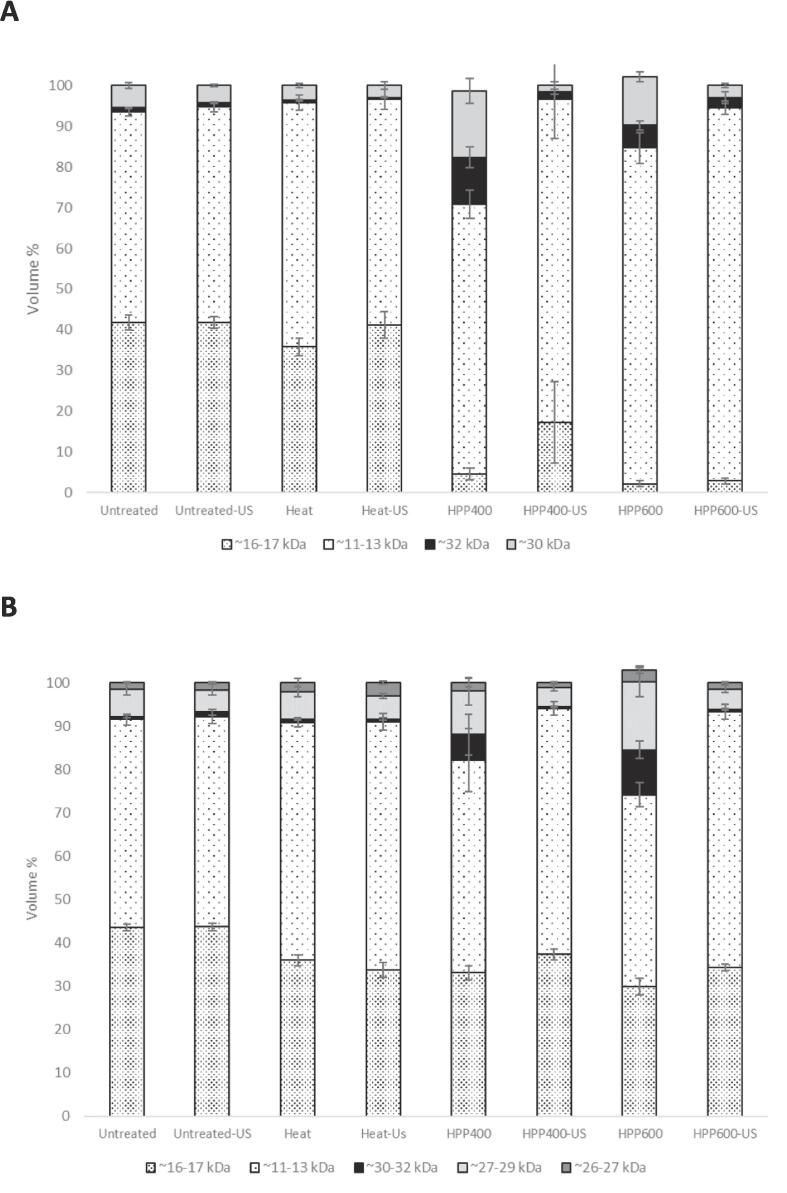
Average relative volume percentage with standard deviation of the two bands detected at ∼16–17 kDa (dotted, dense) and ∼11–13 kDa (dotted, light) respectively in Native-PAGE of serum phase of untreated and treated samples upon pH adjustment.

**Fig. 7:**
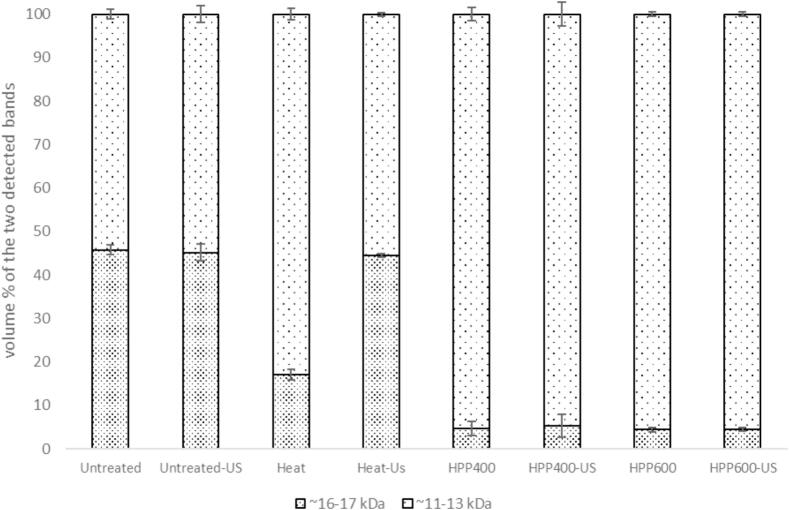
Average relative volume percentage with standard deviation of the five and four bands detected in Native- (A) and SDS-PAGE (B) at ∼30–32 kDa, ∼27–29 kDa and ∼26–27 kDa respectively when the serum phase of untreated and treated sample were subjected to the respective analysis upon ultracentrifugation.

The serum phase of untreated and treated sample obtained upon ultracentrifugation was subjected to Native- (Fig. S1) and SDS-PAGE (Fig. S2) and semi-quantitative protein determination (Fig. 5). Three bands at ∼30–32 kDa, ∼27–29 kDa and ∼26–27 kDa were detected in SDS-PAGE of the sample serum phase (Fig. 6), attributed to α-CN, β-CN and κ-CN respectively. The band assignment was based on Anema and Klostermeyer [19] corresponding to the band profile of untreated sample which was not subjected to ultracentrifugation (Fig. S2, lane 2 and 11). Native-PAGE of serum phase from ultracentrifuged sample (Fig. S1) showed two bands at ∼32 kDa and ∼30 kDa, while there was no visible band within this range when Native-PAGE was performed on the serum phase of the sample upon pH adjustment (∼pH 4.6) (Fig. S1). In addition, two bands at ∼16–17 and ∼11–13 kDa were detected in Native- (Fig. S1) and SDS-PAGE (Fig. S2) of the serum phase obtained after ultracentrifugation and pH adjustment. These bands were attributed to β-Lg and α-La respectively [19]. In total, there were four and five bands identified in Native- and SDS-PAGE upon ultracentrifugation respectively, while Native-PAGE of sample serum phase following pH adjustment yielded two bands. Relative to the total band volume of all the bands identified, the percentage (%) of each band’s volume was calculated. Fig. 6A and B present the average volume % of the respective bands identified in Native- and SDS-PAGE of serum phase obtained upon ultracentrifugation respectively. Fig. 7 shows the volume % of the two bands detected in Native-PAGE of serum phase after pH adjustment.

As for the untreated sample, the volume % of the β-Lg and α-La bands at ∼16–17 and ∼11–13 kDa respectively accounted for ∼90–95 % of the total volumes of all the bands identified in Native- and SDS-PAGE upon ultracentrifugation (Fig. 6A and B). Following heat treatment, the volume % of the band at ∼16–17 kDa in the serum phase showed a decrease in both Native- and SDS-PAGE, indicating loss of native- and native-like β-Lg from the serum phase [39] and increased incorporation of thermally denatured β-Lg in the milk curd at pH adjustment (Fig. 7). The remaining intensity at ∼16–17 kDa upon pH adjustment (Fig. 7) also indicated that there was a fraction of native- and native-like β-Lg in the serum phase after heat treatment which remained unbound with casein micelles. In addition, heat treatment decreased the protein concentration of the serum phase (Fig. 5) indicating increased sedimentation of denatured β-Lg and CN fractions through thermally induced complexation [29], while this did not affect the overall average particle size of the heat-treated sample (Table 1).

Upon HPP 400 MPa, there was a large increase in the band volume % of the bands at ∼30–32 kDa in Native-PAGE of serum phase upon ultracentrifugation (Fig. 6A). This increase indicated increased proportion of native, and native-like CN fractions in the serum phase through HPP-induced dissociation and dispersion of CN fractions including κ-CN and α-CN [29]. The band volume % of the bands at ∼30–32, ∼27–29, and ∼26–27 kDa in SDS-PAGE similarly increased (Fig. 6B). The HPP dispersion of the CN fractions was accompanied by the increased protein concentration of the serum phase (Fig. 5). These results corresponded with the increased concentration of non-sedimental (serum) caseins in reconstituted skim milk following ultra-high-pressure homogenization [40]. The authors attributed the increase to the particle size reduction of fragmented casein micelles which were too small to sediment upon ultracentrifugation. Similar decrease in particle size was observed in the samples subjected to HPP 400 and 600 MPa (Fig. 1). The decreased band volume % at ∼16–17 kDa in relation to that at ∼11–13 kDa in Native-PAGE (Fig. 7) indicated that α-La was more resistant to pressure treatment [29].

HPP 600 MPa showed a further decrease in the band volume % at ∼16–17 kDa in Native −PAGE (Fig. 6A) indicating more extensive denaturation of pressure-sensitive β-Lg and subsequent loss of native- and native-like β-Lg fractions from the serum phase by the higher pressure. The band volume % of the CN bands (∼30–32 kDa) in Native-PAGE was also lower than that of HPP 400 MPa, indicating a decrease in the proportion of native- and native-like CN fractions in the serum phase after HPP 600 MPa. However, SDS-PAGE (Fig. 6B) showed a larger total volume % of the CN bands (at ∼26–32 kDa) after HPP 600 MPa implying that the higher pressure dispersed more proteins in the serum phase in total, but a larger proportion of these dispersed fractions were in non-native, denatured form. This, along with the further reduction in particle sizes (Fig. 1) may have accounted for the slightly higher average protein concentration of serum phase at HPP 600 MPa as compared to HPP 400 MPa (Fig. 5).

Interestingly, US following HPP at 400 and 600 MPa decreased the protein concentration of the serum phase (Fig. 5). This was accompanied by a decrease in the band volume % of the CN bands at the range ∼26–32 kDa in both Native- and SDS-PAGE (Fig. 6A and B) demonstrating the main involvement of the CN fractions detected at ∼26–32 kDa in promoting the sedimentation. There was a corresponding increase in the total volume % of the whey protein bands at ∼16–17 and ∼11–13 kDa in Native- and SDS-PAGE by the intensity loss of the CN bands upon US. However, the band at ∼16–17 kDa in Native-PAGE (Fig. 6A) remained low when US followed HPP 600 MPa, while there was an increase in the intensity though with large variations upon US after HPP 400 MPa. Considering the similar intensity increase in SDS-PAGE for both treatments (Fig. 6B), these results indicated that there was a larger proportion of native, native-like β-Lg fractions in the serum phase when US followed HPP 400 MPa. Moreover, the pressure-dependent difference was observed when the serum phase was obtained by ultracentrifugation but not by pH adjustment. This implied that these HPP-dispersed, native-like β-Lg fractions after US and HPP 400 MPa were bound to the micelles and lost from the serum phase by pH adjustment, while ultracentrifugation allowed these fractions to be remained in the serum phase [41], perhaps due to the small particle sizes (Fig. 1) [40].

US treatment of heat-treated sample also increased the volume % of the band at ∼16–17 kDa in Native-PAGE upon ultracentrifugation (Fig. 6A), while this was most evident upon pH adjustment (Fig. 7). This indicated that US increased a proportion of native- and native-like β-Lg left in the serum phase that were unbound to the micelles and not incorporated in the curd upon pH adjustment. This could be related to a breakup and dissociation of thermally induced whey-κ-CN complex by US as reported by Chandrapala et al. [5], and implied that the originally micelle-bound β-Lg fractions emerged into native-like serum subunits through US, although it did not affect the particle size (Fig. 1). Unlike US effect on HPP treated sample, however, there was little change in the protein concentration of the serum phase upon US of heat-treated sample when subjected to ultracentrifugation (Fig. 5). Considering the minor US effect on the CN bands (Fig. 6), these results indicated the little involvement of the CN fractions and sedimentation when US was applied to the heat-treated sample.

The little US-dependent variation observed in the intensity bands of the untreated sample in Native- or SDS-PAGE, and regardless of whether the serum phase was obtained upon ultracentrifugation or pH adjustment (Fig. 6, Fig. 7) indicated that the observed US effect was limited to the serum fractions that had emerged through the preceding heat- and HPP treatments, with the main involvement of the CN- and β-Lg fractions respectively. These process-dependent effects of US in the intensity bands of the serum phase corresponded well with those observed for the surface hydrophobicity (Fig. 3) and total and surface SH contents (Fig. 4) of the untreated and treated samples subjected to US. It is possible that US interference on untreated sample as seen in the total and surface SH groups (Fig. 4) may have occurred mainly to the sedimentable fractions and / or fractions bound to the micelles thus undetected by the electrophoresis analyses of the serum phase.

## 4 Conclusions

In conclusion, the US effect on reconstituted skim milk when applied at 68 kHz, 500 W below denaturation temperature (∼30 °C) varied by the preceding thermal- and non-thermal (HPP) processing. Heat (85 °C, 20 min) and HPP at 400 and 600 MPa, 15 min resulted in varying degrees of denaturation and conformational changes and as a result, the following US treatment demonstrated intensity-dependent exposure (unfolding) and burial (renaturation) of surface reactive sites respectively. HPP intensity influenced the susceptibility of surface reactive sites for US interference, as seen by the effect difference on surface hydrophobicity and surface SH group following HPP 400 and 600 MPa. Furthermore, US following HPP promoted sedimentation of HPP-dispersed serum CN fractions, while US following heat treatment was directed at the β-Lg originally bound to the micelles, which then emerged as native-like, unbound subunits. Moreover, the US-induced physical change was largely limited to the breakup of the large aggregates formed upon HPP, indicating that the primary US effect when applied under the given conditions was at the molecular level.

## CRediT authorship contribution statement

**Oluyemi Oriomah:** Writing – review & editing, Investigation, Formal analysis, Data curation. **Estefanía Noriega Fernández:** . **Izumi Sone:** Writing – review & editing, Writing – original draft, Supervision, Methodology, Investigation, Formal analysis, Data curation, Conceptualization.

## Declaration of competing interest

The authors declare that they have no known competing financial interests or personal relationships that could have appeared to influence the work reported in this paper.
