# Supplementary figures and images for: Prior thermal and high-pressure processing alters the impact of high intensity ultrasound on reconstituted skim milk
Source: Ultrason Sonochem. 2024 Jul 22;109:107000. doi: 10.1016/j.ultsonch.2024.107000 (PMC11338986; doi:10.1016/j.ultsonch.2024.107000)

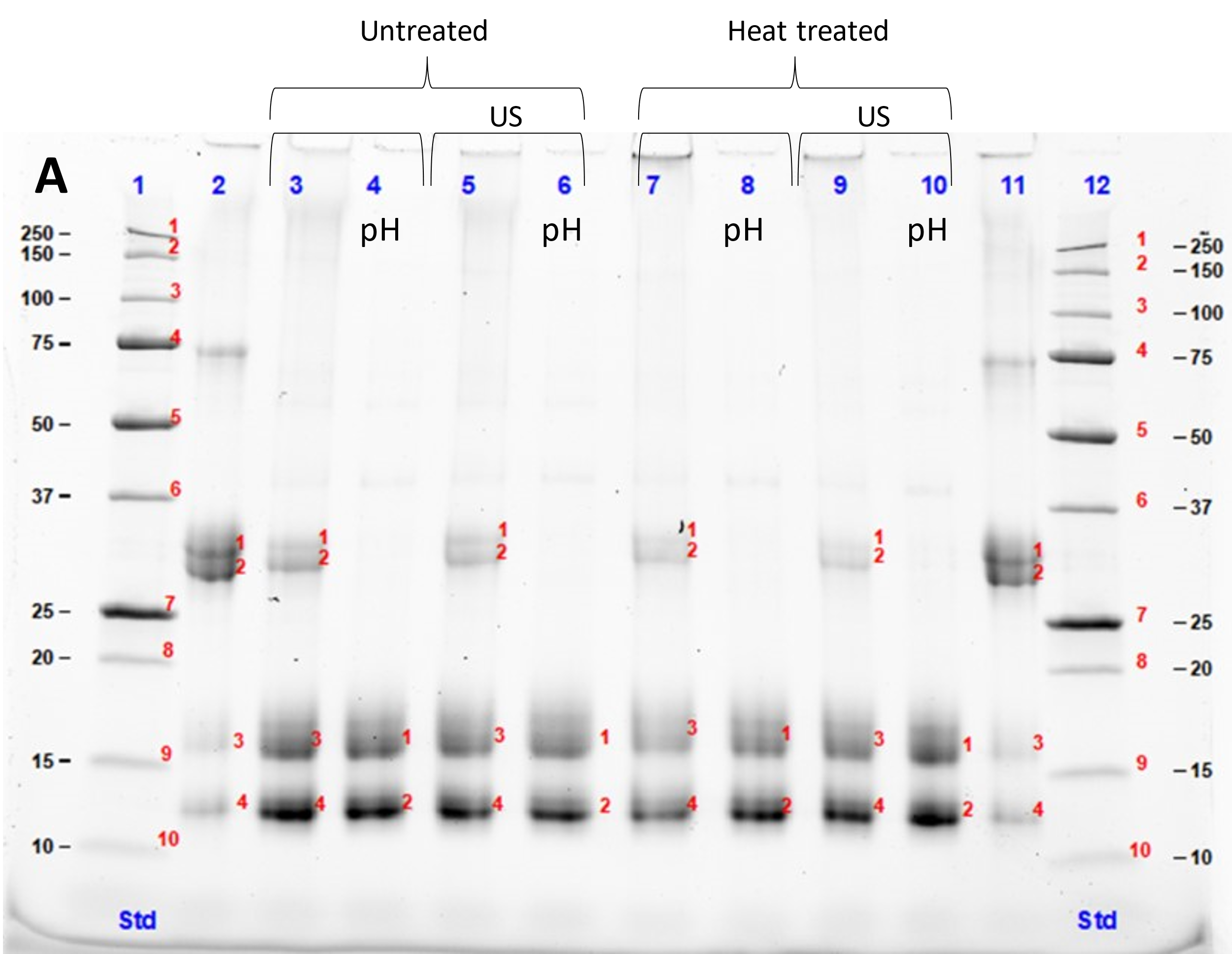


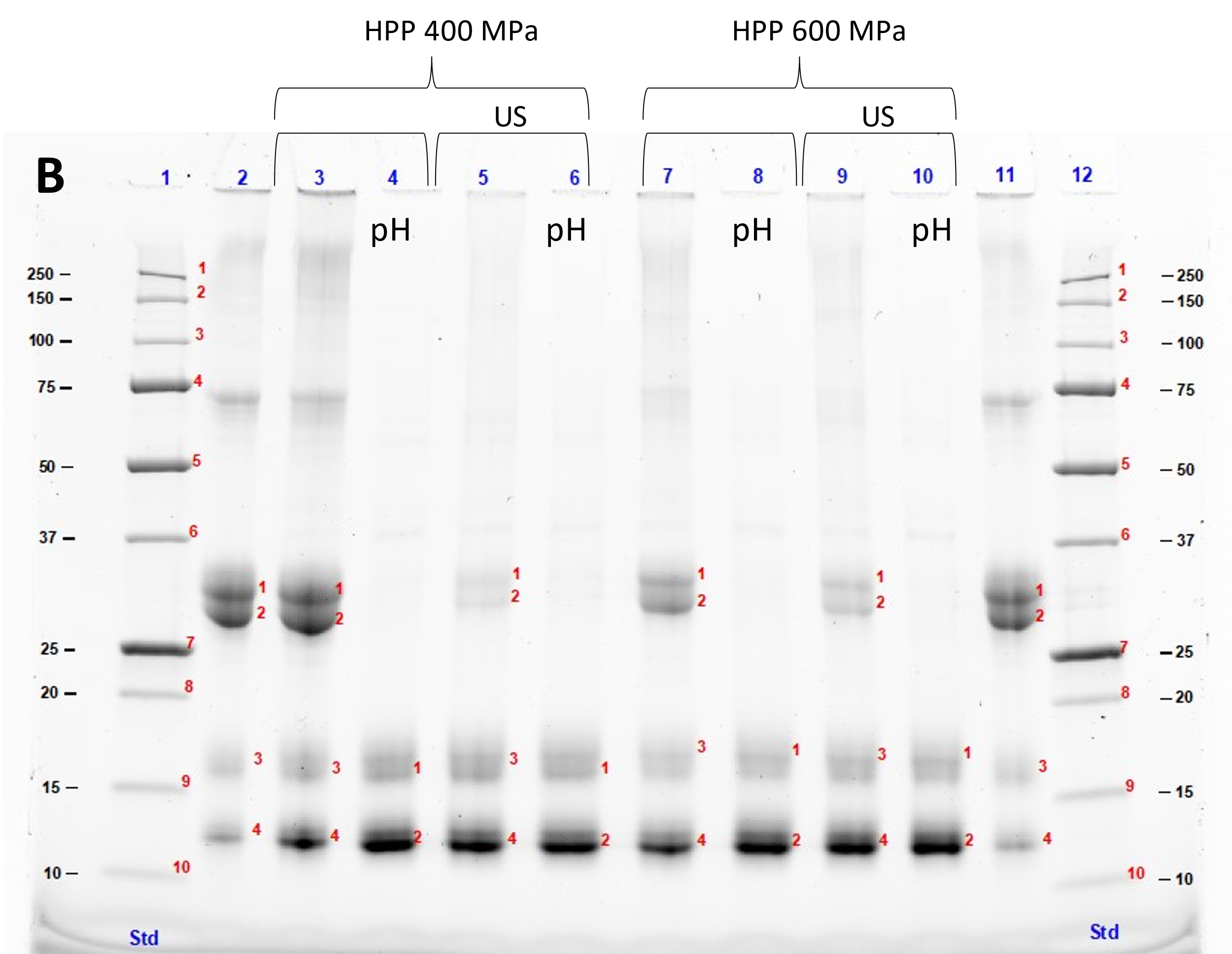

Supplement: Supplementary Data 1 [file mmc1.docx]

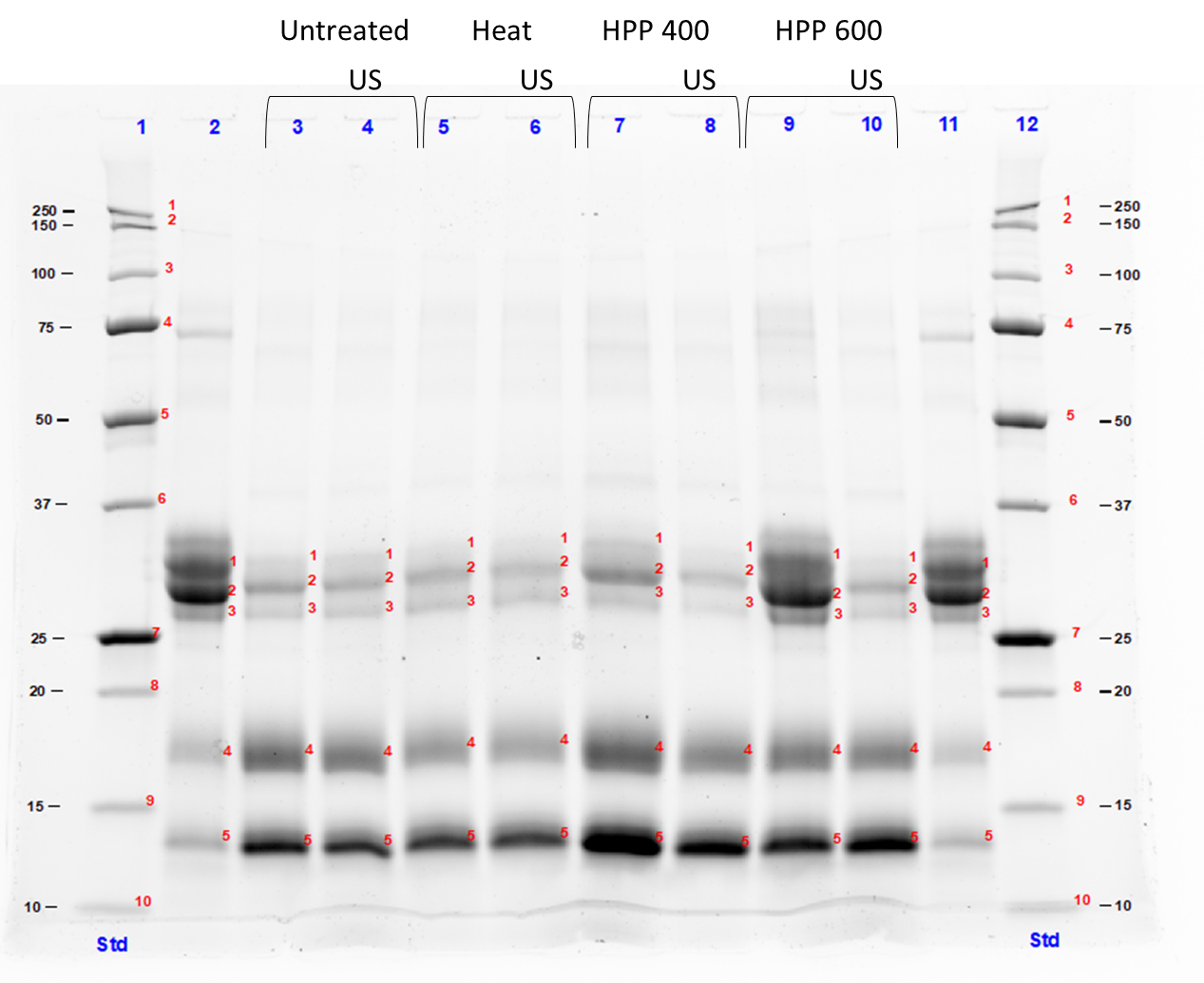

Supplement: Supplementary Data 2 [file mmc2.docx]
